# Improving Accuracy of Lung Nodule Classification Using Deep Learning with Focal Loss

**DOI:** 10.1155/2019/5156416

**Published:** 2019-02-04

**Authors:** Giang Son Tran, Thi Phuong Nghiem, Van Thi Nguyen, Chi Mai Luong, Jean-Christophe Burie

**Affiliations:** ^1^ICTLab, University of Science and Technology of Hanoi, Vietnam Academy of Science and Technology, 18 Hoang Quoc Viet, Cau Giay, Hanoi, Vietnam; ^2^Sorbonne Université, IRD, UMMISCO, Unité de Modélisation Mathématiques et Informatique des Systèmes Complexes, F-93143 Bondy, France; ^3^Medical Science and Technology Laboratory, University of Science and Technology of Hanoi, Vietnam Academy of Science and Technology, 18 Hoang Quoc Viet, Cau Giay, Hanoi, Vietnam; ^4^Department of Radiology, Vietnam National Cancer Hospital, Hanoi, Vietnam; ^5^Institute of Information Technology, Vietnam Academy of Science and Technology, 18 Hoang Quoc Viet, Cau Giay, Hanoi, Vietnam; ^6^L3i Laboratory, University of La Rochelle, La Rochelle, France

## Abstract

Early detection and classification of pulmonary nodules using computer-aided diagnosis (CAD) systems is useful in reducing mortality rates of lung cancer. In this paper, we propose a new deep learning method to improve classification accuracy of pulmonary nodules in computed tomography (CT) scans. Our method uses a novel 15-layer 2D deep convolutional neural network architecture for automatic feature extraction and classification of pulmonary candidates as nodule or nonnodule. Focal loss function is then applied to the training process to boost classification accuracy of the model. We evaluated our method on the LIDC/IDRI dataset extracted by the LUNA16 challenge. The experiments showed that our deep learning method with focal loss is a high-quality classifier with an accuracy of 97.2%, sensitivity of 96.0%, and specificity of 97.3%.

## 1. Introduction

Lung cancer is one of the most serious and common types of cancer all over the world, both in number of new patients and in number of fatalities. There were estimated to be 1.8 million new cases of lung cancer worldwide in 2015 [1]. In the US in 2017, a total of 225,000 lung cancer new cases and 155,870 deaths are listed, accounting for 26% total number of cancer-related deaths [1]. According to Torre et al. [2], an amount of 55% of patient's survival rate of lung cancer can be achieved with localized (early) stage detection. Due to this, it is necessary to examine and observe lung nodules closely when they are still at the early stage.

Computed tomography (CT) exam is one of the most effective ways for early stage detection of lung nodules due to its ability to construct 3D images of the chest, producing high resolution of nodules and tumor pathology. However, early detection of lung nodules is a difficult and time-consuming task: radiologists have to manually and carefully analyze a large number of images in CT scans. Doi [3] shows that radiologists may miss up to 30% of lung nodules due to overlaps between them and other normal anatomic structures.

Computer-aided diagnosis (CAD) systems are designed to aid doctors and radiologists in lung nodule detection and diagnosis. The process of CAD for lung cancer generally consists of a detection system (CADe) and an aided diagnostic system (CADx). The goal of CADe is to divide the pulmonary nodule candidates as nodules or nonnodules (e.g., normal anatomic structures), while CADx aims at categorizing the detected nodules as benign or malignant nodules [4]. Due to its design, accuracy of lung nodule classification is vital for the success of a CADe system.

Nowadays, deep learning (a variant of neural networks with more hidden layers than the traditional counterpart) is considered as one of the best solutions to many problems of computer vision and pattern recognition such as image analysis, speech recognition, natural language processing, etc. Convolutional neural network (CNN), a type of neural networks using convolution operators in its layer, is widely applied in object detection and classification with an amazing performance in terms of accuracy. This advantage of deep learning makes it potential for lung nodule detection and classification systems.

In this paper, we study the problem of classifying pulmonary nodule candidates in CT images as nodule or nonnodule. Our objective is to enhance precision of CADe systems by exploiting the advantage of deep learning. In detail, we propose a new 2D deep convolutional neural network architecture with the use of focal loss [5] for boosting classification accuracy. We will show that our CNN network with focal loss is a high-quality method for lung nodule classification.

The rest of the paper is organized as follows. We briefly review related works in Section 2.  Section 3 describes the materials and methods used to classify pulmonary nodule candidates as nodule or nonnodule. Experiments and discussions are presented in Section 4. Finally, Section 5 concludes our work and presents possible future directions.

## 2. Related Works

CNN started as an evolution of artificial neural network by using convolution operators to achieve better understanding of the input image. This architecture is designed based on the biological visual system and thus is very effective for image recognition problems, regardless of size or scale. In such an architecture, each neuron is linked to another in a way such that it responds to the receptive field around it. Additionally, since each neuron is only connected to several “nearby” other neurons, the amount of weights for a CNN is much less than a fully connected (FC) network. Therefore, CNN produces better accuracy for image recognition while taking less time for training than FC networks. Since its inception [6], CNN has been “deepened” with many proposed network architectures. For example, AlexNet [7] has 8 layers with 1000 classes; VGG [8] has 25 layers; and ResNet [9] consists of up to 152 layers.

Exploiting the advantages of deep learning, many methods are introduced to increase classification accuracy of lung nodule candidates in CT scans. Li et al. [10] proposed a 2D deep convolutional neural network to categorize pulmonary nodule candidates as nodule or nonnodule. The proposed network was trained and validated on 62,492 regions-of-interest (ROI) image patches extracted from 1,010 CT scans of the LIDC/IDRI dataset. 40,772 out of 62,492 patches are lung nodules and 21,720 out of 62,492 patches are nonnodules. By experiments, the method is able to gain up to 86.4% accuracy and 89.0% sensitivity of lung nodule classification.

Kuruvilla and Gunavathi [11] proposed a computer-aided method for classification of lung cancer in CT images. The authors used the statistical features such as mean, standard deviation, skewness, kurtosis, fifth central moment, and sixth central moment as features for classification. The feed forward back propagation neural network is proved to give better classification results than the feed forward neural network. The experiments demonstrated that the method obtains an accuracy of 93.3%, a sensitivity of 91.4%, and specificity of 100%. These results show the high quality of the method in determining noncancerous nodules correctly.

Choi and Choi [12] introduced a pulmonary nodule detection method using hierarchical block classification. The method firstly divides the image into three-dimensional blocks. The entropy analysis is then applied to select informative blocks for identifying nodule candidates. Finally, support vector machine (SVM) is used to classify nodule candidates as nodule or nonnodule. The evaluation on the LIDC dataset shows that the method is able to achieve a very good accuracy of 97.6%, a sensitivity of 95.2%, and a specificity of 96.2%.

Setio et al. [13] applied multiview convolutional networks (ConvNets) to identify lung nodule candidates. The method was trained on 1,186 nodules extracted from the LIDC/IDRI dataset by the LUNA16 challenge. The experiments show that the method gains a sensitivity of 90.1%. Torres et al. [14] used a feed forward neural network (FFNN) as a nodule candidate classifier. The network configuration includes 13 input neurons, 1 hidden layer with 25 neurons, and 1 neuron in the output layer to present the binary classifier. The training and testing candidates were performed on 1,018 CT scans from the LIDC/IDRI dataset. By the experimental results, the method obtains a sensitivity up to 89.1%.

The LUNA16 challenge [15] proposed an evaluation framework for automatic nodule detection algorithms using the largest publicly available reference database of CT scans, the LIDC-IDRI dataset [16]. The challenge extracted 1,186 lung nodules from LIDC-IDRI chest CT images and provided these nodules as positive candidates for researchers. The outcomes of the challenge shows that the best individual detection system can obtain up to 92.9% sensitivity of lung nodule detection.

## 3. Materials and Methods

### 3.1. Dataset

The dataset used in this paper is extracted from the LIDC/IDRI dataset [16] by the LUNA16 challenge [15]. We inherit the extracted dataset of the LUNA16 challenge since it fits with our objective of classifying pulmonary nodule candidates in CT images as nodule or nonnodule. LIDC/IDRI is the largest publicly available reference database of chest CT scans. It consists of 1,018 thoracic CT scans collected from seven academic institutions with a wide range of scanner models and acquisition parameters [16]. CT images from each scan is associated with an XML file recording nodule reports and diagnosis reports of image annotation process from four experienced thoracic radiologists.

Each LIDC/IDRI annotation was created by a two-phase reading process. In the first blinded reading phase, suspicious lesions were independently annotated by four experienced thoracic radiologists as nodule >3 mm, nodule >3 mm, or nonnodule (any other pulmonary abnormality). In the second nonblind reading phase, the blinded results of all other radiologists were revealed to each radiologist, who then decided to accept or reject each annotation. No consensus was forced. The LIDC/IDRI annotations for nodules ≥3 mm include position, diameter of nodule in each CT slice, and subjective ratings on a five-point or six-point scale of the pathologic features: calcification, internal structure, subtlety, lobulation, margins, sphericity, malignancy, texture, and spiculation [10].

As suggested by other research studies [17, 18], thin-slice CT scans should be useful for pulmonary nodule management. Due to this, from 1,018 cases, the LUNA16 challenge discarded the CT scans having a slice thickness greater than 3 mm and having inconsistent slice spacing or missing slice. As a result, 888 CT scans are extracted from 1,018 cases of the LIDC/IDRI dataset.

In 888 CT scans, there are 36,378 annotations marked by the radiologists. Since nodules <3 mm or nonnodule lesions are not considered in lung cancer screening protocols, only nodules ≥3 mm are extracted by the LUNA16 challenge. Besides, one annotation could be marked by more than one radiologist. Due to this, the annotations from more than one radiologist for one nodule were merged if their locations are closer than the sum of their radii. With merged annotations, positions and diameters are averaged. The result of this filtering process is a set of 2,290, 1,602, 1,186, and 777 nodules marked by at least 1, 2, 3, and 4 radiologists, respectively [15].

To ensure the quality of extracted nodules, the challenge considered only the nodules marked by a majority of the radiologists (at least 3 out of 4 radiologists) as the positive candidates to detect lung nodules. From this, the challenge obtained 1,186 lung nodules extracted from the LIDC/IDRI dataset as positive candidates for pulmonary nodule detection and classification. For negative candidates, the challenge used three algorithms [19–21] to generate the locations of nonnodules, presenting any other pulmonary lesions from 888 CT scans. This results in 549,879 negative candidates.

In our study, we use 1,186 positive candidates and 549,879 negative candidates provided by the LUNA16 challenge and classify them as nodule or nonnodule. Since the number of positive and negative candidates are extremely imbalanced for training and testing the model, we counter this imbalance by using data augmentation and focal loss function, as will be described in the next sections.

### 3.2. Data Augmentation

Normally, a large number of positive and negative samples are necessary to satisfy the training step of convolutional neural networks. Due to this, we apply the following augmentation techniques to generate more lung nodules for training the model:

- Rotating from −25° to 25° with a 5° step
- Flipping horizontally
- Flipping vertically
- Flipping both horizontally and vertically

We choose not to apply nonuniform transformations, such as stretching or skewing, due to the importance of nodule's shape for detection process. After augmentation, we have 16,189 positive patches (those that are too close to the slice's edges are discarded).

### 3.3. Focal Loss

Initial goal of *focal loss* function proposed by Lin et al. [5] is to address the problem of extreme balance between foreground and background classes during training in object detection scenarios. Focal loss is mainly used for object detection, but we also show that its sparse-specific characteristics are also applicable for classification problem with imbalanced dataset.

The starting point of focal loss is the cross-entropy loss function [22] for binary classification, defined as

(1) $$\begin{array}{c} \text{CE}\left(p,y\right)=\left\{\begin{array}{c} -\log\left(p\right),\;\text{if }\;\;y=1,\\ -\log\left(1-p\right),\;\text{otherwise}, \end{array}\right. \end{array}$$

in which *y* ∈ {−1,1} denotes the ground truth for negative and positive classes, respectively, and *p* ∈ [0,1] indicates the model's estimated probability for the class with label *y*=1. The authors argue that cross-entropy loss exhibits a loss with nontrivial magnitude even with easily classified samples (i.e., *yp*+(1 − *y*)(1 − *p*) ≫ 0.5). Therefore, these small loss values, accumulated with a large number of easy samples, can easily surpass the rare class.

For simplicity, let

(2) $$\begin{array}{c} p_{t}=\left\{\begin{array}{c} p,\;\text{if}\;\;y=1,\\ 1-p,\;\text{otherwise}. \end{array}\right. \end{array}$$

In order to balance the importance of positive/negative samples, a weighting factor *α* ∈ [0,1] is introduced in a similar notation:

(3) $$\begin{array}{c} \alpha_{t}=\left\{\begin{array}{c} \alpha ,\;\text{if}\;\;y=1,\\ 1-\alpha ,\;\text{otherwise}. \end{array}\right. \end{array}$$

For reducing the loss contribution from easily classified samples, a modulating factor *m* with a tunable focusing parameter *γ* ≥ 0 is introduced to the cross-entropy loss:

(4) $$\begin{array}{c} m={\left(1-p_{t}\right)}^{\gamma }. \end{array}$$

Taking these two new factors into equation (1), the proposed focal loss function becomes

(5) $$\begin{array}{c} \text{FL}\left(p_{t}\right)=-\alpha_{t}{\left(1-p_{t}\right)}^{\gamma }\text{ log}\left(p_{t}\right). \end{array}$$

Note that *α* and *γ* are two parameters indicating how sensitive it is to the easily classified samples. In this work, we propose to apply this focal loss function to the end of our proposed 2D CNN architecture. We will show that this proposal helps in increasing accuracy of lung nodule classification in CAD systems.

### 3.4. Construction of CNN Architecture

In this work, we propose a new 2D deep CNN architecture, namely, LdcNet, so as to improve classification accuracy of pulmonary lesions as nodule or nonnodule. LdcNet consists of 3 different layer types: convolutional layer, pooling layer, and fully connected layer. A *convolutional layer* performs 2D convolution operators on each input image. These layers can extract features from the input image during training phase. Deeper layers can detect higher level of abstraction from features. A *pooling layer* works on individual feature channels and aggregates nearby feature values into one. Therefore, it reduces the number of trainable parameters, controls overfitting, and effectively shortens the training time. A *fully connected layer* links all neurons in the current layer with all neurons from previous layer. For 2D images, fully connected layers work less effectively than convolutional layers due to the nature of spatial relation in images. Fully connected layers also increase number of trainable parameters in a network and therefore, lengthen time required for training. *Dropout*, a regulation technique to reduce number of neurons and connections, is proposed to solve such problem.

**Figure 1:**
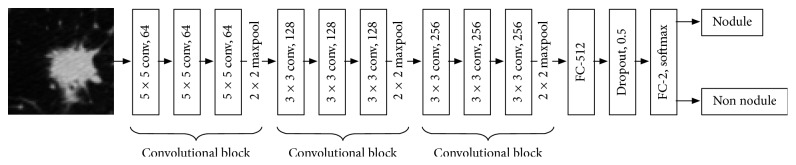
Our proposed 2D deep CNN architecture (LdcNet).

Figure 1 depicts our proposed 2D deep CNN architecture. The input images, having size 64 × 64 in grayscale format, are fed into the network. Our network consists of 15 layers, 9 of which are convolutional layers. We use 3 convolutional blocks; each consists of 3 convolutional layers and one pooling layer using max operator. The later blocks extract more features on higher abstraction level than those at the beginning of the network (64, 128, and 256 features, respectively). The first block uses 5 × 5 filter and the later ones use 3 × 3 filters. We will justify the number of convolutional blocks in Section 4. Each convolutional layer is followed by a ReLU (rectified linear unit) activation function [7] due to its efficiency in computing performance when compared with other nonlinear activation functions. ReLU is defined as

(6) $$\begin{array}{c} y=\left\{\begin{array}{c} x,\;\text{if}\;\;x\geq 0,\\ 0,\;\text{otherwise}. \end{array}\right. \end{array}$$

Or, in other words, *y*=max(0, *x*).

The last two fully connected layers aim at solving the classification problem from the extracted features from the previous convolutional blocks. They are followed by a ReLU and a *softmax* activation function, respectively. Softmax is a normalized exponential function that produces categorical distribution from the previous layer's output. In our network, softmax is used to calculate nodular probability for each prediction. We apply dropout strategy after the first fully connected layer to reduce overfitting.  Table 1 details the deep CNN configuration, including input and output dimensions, that we propose in this paper. Note that the first (#1) and the last 3 convolutional layers (#9–#11) are padded to produce large output size after the first 3 convolutional blocks, in order to keep the network deep.

## 4. Experiments and Results

### 4.1. Experimental Setup

#### 4.1.1. Preprocessing

**Figure 2:**
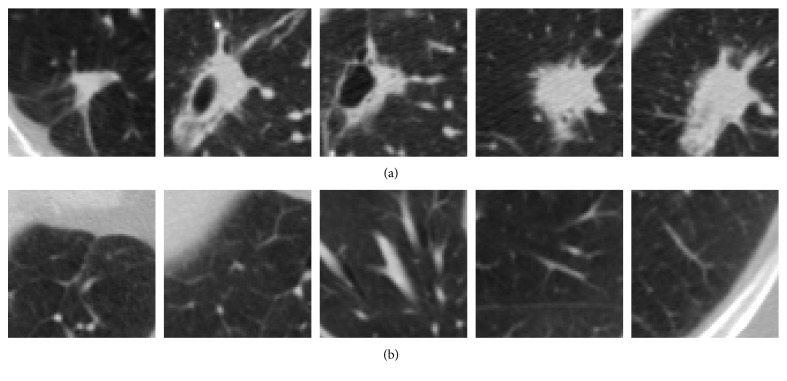
Extracted patches for positive samples (a) and negative samples (b).

We firstly perform data preprocessing to standardize the image data. Each slice is loaded and converted to grayscale image. We extract one nonoverlapping image patch of size 64 × 64 pixels for each annotation of both positive and negative samples. The annotations which are too close to the edges of slices (less than 32 pixels) are removed.  Figure 2 illustrates some extracted image patches using this method. The average number of patches per scan of the whole dataset is 620.5. The extracted image's intensity *I* is then scaled to Hounsfield unit *I*_HU_ ∈ [−1000,400] and linearly normalized to *I*_norm_ ∈ [0,1] range using the transformation *I*_norm_=(*I*_HU_+1000)/1400 before being used as input for training the model.

The dataset is divided into training set (60%), validation set (20%), and test set (20%). Note that the test set only contains samples from the original dataset, not augmented data.

#### 4.1.2. Performance Metrics

Accuracy (ability to differentiate the nodule and nonnodule cases correctly), sensitivity (ability to determine the nodule cases correctly), and specificity (ability to determine the nonnodule cases correctly) are used to measure the correctness of the classification. These metrics are widely used in binary classification problems and are defined as follows:

(7) $$\begin{array}{c} \text{accuracy}=\frac{\text{TP}+\text{TN}}{\text{TP}+\text{TN}+\text{FP}+\text{FN}},\\ \text{sensitivity}=\frac{\text{TP}}{\text{TP}+\text{FN}},\\ \text{specificity}=\frac{\text{TN}}{\text{TN}+\text{FP}}, \end{array}$$

where TP (true positive) represents the number of cases correctly identified as nodules; FP (false positive) represents the number of cases incorrectly identified as nodules; TN (true negative) represents the number of cases correctly identified as nonnodules; and FN (false negative) represents the number of cases incorrectly identified as nonnodules.

#### 4.1.3. Hardware and Software Configuration

We perform our experiments on two different servers. The training task (with hyperparameter exploration and tuning), which consumes a lot of time, is performed on a dual-socket Intel Xeon 2620 v3 (12 cores, 24 threads in total), 128 GB of DDR4 memory, and 4 NVIDIA Tesla K80 (8 GPUs in total). This server is chosen for hyperparameter exploration since each set of hyperparameters can be trained in parallel on a single GPU; thus 8 different network configurations can be trained, validated, and tested at the same time.

For CAD inference after training, we use another local server, having an Intel Xeon 2620 v3 (6 cores, 12 threads at 2.4 GHz), 32 GB of DDR4 memory, and an NVIDIA GeForce GTX 1080 (2560 CUDA cores and 8 GB of memory). This selection is due to the better inference speed of a single GTX 1080 when compared with a Tesla K80.

We implement our CAD system using Keras 2.1.3 with TensorFlow 1.3 as the backend, along with CUDA 8.0 for GPU acceleration. Our CAD system is implemented with Python 2.7 and runs on Debian Jessie 9.4.

#### 4.1.4. Training

During the training phase, we use Adam optimizer [23] with learning rate lr=0.0001 without exponential decay, and default parameters *β*_1_=0.9, *β*_2_=0.999 as proposed by Kinga et al. [23]. Each evaluation consists of 100 training epochs and a prediction on test set. Various hyperparameters, such as batch size or focal loss *γ* and *α*, will be explored in Section 4.2.

#### 4.1.5. Testing

During the testing phase, the *k*-cross validation method is used. This method splits the data into *k* parts of the same size. The evaluation is performed in *k* iterations; each uses one different part for testing, while the rest is used for training. In our evaluation, we use *k*=10 as a popular choice of this validation method. Besides, we eliminate all augmented data from positive samples in each testing part to get results that are more accurate.

### 4.2. Results and Discussions

#### 4.2.1. Hyperparameter Tuning

It is of importance to find the best configuration for our proposed network, including network architecture (number of convolutional blocks) and hyperparameter values. In this part, we justify the choice of such configuration using a set of evaluations and tuning our network.

To obtain the best set of parameters, we vary each of them and then perform training and predicting on our network. The first parameter is input scale. Although the patches are extracted with 64 × 64 pixels, we vary their scale to feed to our CNN from 0.3125 to 1. For example, a scale of 0.875 produces a smaller patch with 56 × 56 pixels. This parameter indicates how well the network interprets and classifies nodules in different resolutions. Secondly, we analyze the effect of training batch size to the output accuracy. We range this parameter from 128 to 256 (as most common CNNs use) in a 32-input step. Thirdly, we try different configurations of the network by altering the number of convolutional blocks in the network. This parameter allows to examine the effectiveness of network depth against binary lung nodule classification accuracy. Finally, we adjust the two parameters of focal loss, namely, *γ* and *α* (experimented in [0.5, 5] and [0.2, 2] ranges with 0.5 and 0.1 steps, respectively), to see how well they behave with the imbalanced dataset of positive and negative samples.

Table 2 summarizes our experimented configurations. The best set of hyperparameters for LdcNet is presented in bold (#7) with accuracy of 97.2%, sensitivity of 96.0%, and specificity of 97.3%. Note that we choose this configuration instead of #8 (with slightly higher accuracy at 97.3%) because of better sensitivity (96.0% vs. 93.3%), which is important for nodule classification problem.

We observe that our network responds well to the test set with big input scale (i.e., input is close to 64 × 64 pixels), large batch size (around 224), and 3 convolutional blocks. The experiments also confirm that *γ* and *α* should be around [1.5, 2.5] and 0.5, respectively, as the authors recommended in [5].

#### 4.2.2. Training Convergence

**Figure 3:**
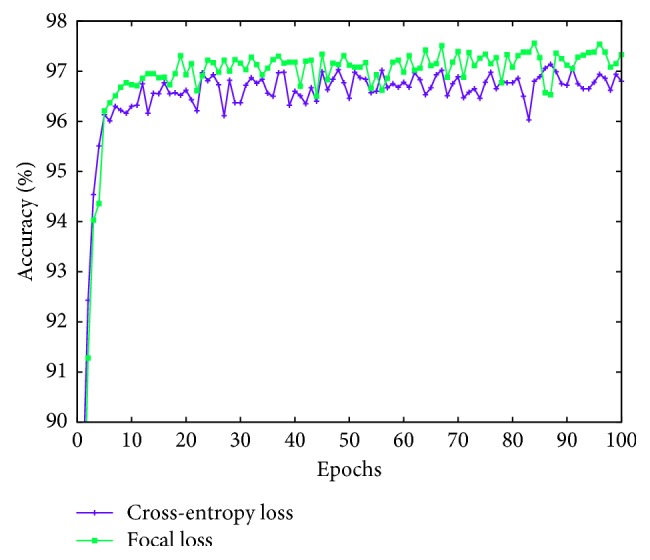
LdcNet's validation accuracy during training with cross-entropy loss vs. focal loss.

Our proposed CNN model using focal loss with the previously selected hyperparameters converges faster than with the popular cross-entropy loss function.  Figure 3 shows our achieved accuracy. It can be seen from this figure that the network stabilizes after around 12 epochs (versus 20 epochs with cross-entropy loss) and achieves slightly better accuracy (average 0.5%–1%).

#### 4.2.3. Effectiveness of Focal Loss

**Figure 4:**
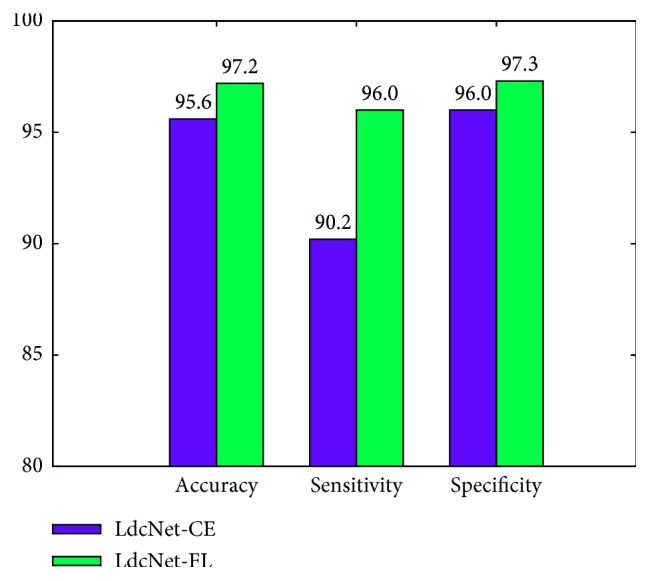
Performance of LdcNet using cross-entropy loss vs. focal loss.

We measure the effectiveness of the focal loss function in lung nodule classification by taking the best result produced by the tuning step and configuring LdcNet with Focal Loss (LdcNet-FL) and cross-entropy loss (LdcNet-CE) accordingly. Obviously, the LdcNet-CE configuration does not use the *γ* and *α* parameters, as these are two exclusive parameters for focal loss.  Figure 4 summarizes our finding in this experiment. We observe that focal loss function can boost the classification's accuracy up to 1.6%, sensitivity up to 5.8%, and specificity up to 1.3% in comparison with cross-entropy loss function.

#### 4.2.4. Receiver Operating Characteristic (ROC) Analysis

**Figure 5:**
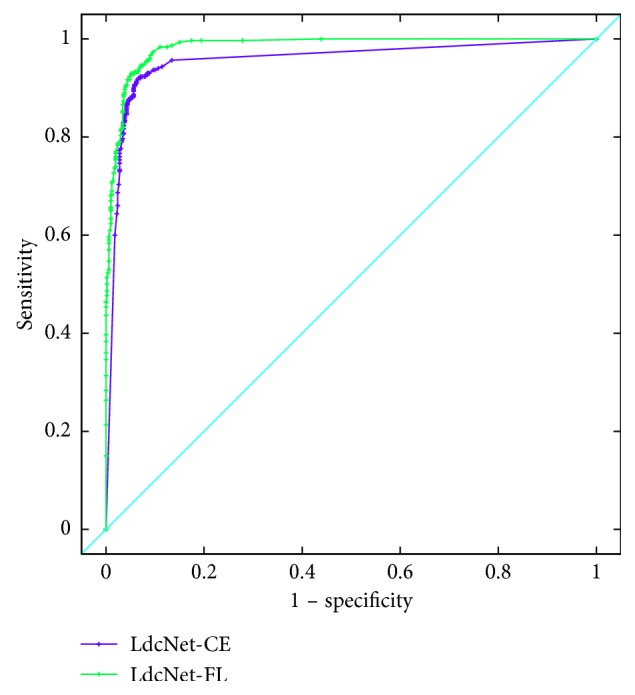
LdcNet's ROC curves with cross-entropy loss vs. focal loss.

ROC curve allows to evaluate performance of a binary classification system by analyzing the relation between true-positive rate (TPR, same as sensitivity) and false-positive rate (FPR, same as 1 − specificity). In detail, TPR and FPR are measured as the classification threshold changes. Each pair of (TPR, FPR) are then plotted to produce a ROC curve. A good classifier generally has high TPR and low FPR; thus its ROC curve would shift toward the top left corner.  Figure 5 shows the ROC curve for our LdcNet, using both focal loss and cross-entropy loss with the classification threshold ranging from 0 to 1 in a 0.01 step. It can be seen from the figure that our LdcNet's ROC curves are very close to the top left corner of the figure, indicating the high quality of our classifiers, especially the LdcNet with focal loss.

#### 4.2.5. Area under Curve (AUC) Analysis

AUC represents the separability, which indicates how well a classification system distinguishes between the positive class and the negative class.  Table 3 summarizes the AUC values of our proposed LdcNet with cross-entropy loss and focal loss. As shown on this table, our approach LdcNet-FL is a high-quality classifier with an AUC value of 98.2%.

#### 4.2.6. Classification Accuracy

To evaluate the correctness of our classifier, we use three metrics: accuracy, sensitivity, and specificity.  Table 4 presents the comparison of our proposed CNN network with other related works in the literature. Strictly speaking, it is hard to compare other works on lung nodule classification since the LIDC dataset changes every year and most of current works do not employ the whole LIDC dataset. From the table, our method has the best sensitivity of 96.0% indicating the high quality of our classifier in determining the pulmonary nodule cases correctly. Besides, although our work has lower accuracy (97.2% vs. 97.6%) and lower specificity (97.3% vs. 100%) than the works in [11, 12], respectively, the consensus level (the number of radiologists agrees with the annotation) used in our work is higher than the ones used in [11, 12]. Generally speaking, our best experimental results (in bold) in Table 4 prove that our CNN method with focal loss is a high-quality classifier with an accuracy of 97.2%, sensitivity of 96.0%, and specificity of 97.3%.

## 5. Conclusion and Perspectives

In this paper, we propose a new 15-layer 2D deep convolutional neural network architecture, namely, LdcNet, to address the problem of classifying pulmonary nodule candidates in CT images as nodule or nonnodule. The network is partitioned into 2 parts: automatic feature extraction with 3 convolutional blocks and classifier with fully connected layers. We apply focal loss function to our proposed network to boost classification accuracy of pulmonary nodules. The evaluation on the LIDC/IDRI dataset extracted by the LUNA16 challenge shows that our deep learning method with focal loss is a high-quality classifier with 97.2% accuracy, 96.0% sensitivity, and 97.3% specificity.

Several research directions can be taken into account to continue this work. Firstly, 3D CNNs can be used to exploit 3D nature of lung nodules from multiple CT slices. When being used with focal loss function, 3D CNNs have potentials to outperform 2D CNNs in terms of accuracy. Secondly, we have yet to apply segmentation techniques of lung volumes to remove out-of-lung areas from extracted patches. Involving such techniques in data preprocessing step can further boost classification accuracy of our proposed deep CNN. Last but not least, in deep learning, classification accuracy often increases when the amount of data used for training increases, thus using larger dataset for training can be a good research direction to continue improving our classification accuracy of lung nodules.

## Figures and Tables

**Table 1:** Detailed configuration of the proposed deep convolutional neural network architecture.

#	Type	Input	Kernel	Output
1	Convolutional	64 × 64 × 1	5 × 5	64 × 64 × 64
2	Convolutional	64 × 64 × 64	5 × 5	60 × 60 × 64
3	Convolutional	60 × 60 × 64	5 × 5	56 × 56 × 64
4	Max pooling	56 × 56 × 64	2 × 2	28 × 28 × 64

5	Convolutional	28 × 28 × 64	3 × 3	26 × 26 × 128
6	Convolutional	26 × 26 × 128	3 × 3	24 × 24 × 128
7	Convolutional	24 × 24 × 128	3 × 3	22 × 22 × 128
8	Max pooling	22 × 22 × 128	2 × 2	11 × 11 × 128

9	Convolutional	11 × 11 × 128	3 × 3	11 × 11 × 256
10	Convolutional	11 × 11 × 256	3 × 3	11 × 11 × 256
11	Convolutional	11 × 11 × 256	3 × 3	11 × 11 × 256
12	Max pooling	11 × 11 × 128	2 × 2	5 × 5 × 256

13	Fully connected	5 × 5 × 256	N/A	512

14	Dropout	512	N/A	512

15	Fully connected	512	N/A	2

**Table 2:** Performance of LdcNet for different architectures and hyperparameter sets.

#	Input and network			Focal loss		Performance (%)		
	Input scale	Batch size	# conv. blocks	*γ*	*α*	Accuracy	Sensitivity	Specificity
1	0.688	128	2	1	1	92.7	77.7	96.2
2	0.594	160	2	1	0.5	94.3	80.3	93.5
3	0.906	256	2	2.5	0.5	94.5	80.1	95.1
4	1.0	224	2	1.5	0.2	95.3	86.7	95.2
5	0.844	224	3	2.5	0.2	97.0	82.7	98.2
6	0.875	256	3	2	0.5	97.2	94.7	97.4
**7**	**0.906**	**224**	**3**	**2.5**	**0.5**	**97.2**	**96.0**	**97.3**
8	0.938	224	3	1.5	0.5	97.3	93.3	97.6
9	0.844	256	4	1.5	0.5	96.8	93.7	97.4
10	0.938	160	4	2	0.7	97.0	85.0	98.1
11	0.844	256	4	1.5	1	97.1	91.3	97.7
12	0.875	128	4	1.5	0.5	97.1	92.0	97.5

**Table 3:** AUC of our proposed LdcNet with cross-entropy loss and with focal loss.

Our LdcNet	Loss function	AUC (%)
LdcNet-CE	Cross-entropy loss	95.6
LdcNet-FL	Focal loss	98.2

**Table 4:** Classification results of LdcNet compared with other works.

Work	# scans	Dataset	Consensus level	Performance (%)		
				Accuracy	Sensitivity	Specificity
**Proposed LdcNet-FL**	**888**	**LIDC/IDRI**	**≥3**	**97.2**	**96.0**	**97.3**
Proposed LdcNet-CE	888	LIDC/IDRI	≥3	95.6	90.2	96.0
Li et al. [10]	1,010	LIDC/IDRI	≥1	86.4	87.1	—
Kuruvilla and Gunavathi [11]	155	LIDC/IDRI	≥2	93.3	91.4	100
Choi and Choi [12]	58	LIDC/IDRI	≥1	97.6	95.2	96.2

## Data Availability

Previously reported medical image data and annotations were used to support this study and are available at https://doi.org/10.1118/1.3528204 and https://doi.org/10.1016/j.media.2017.06.015, respectively. These prior studies (and datasets) are cited at relevant places within the text as references [15, 16].

## References

[1] Siegel R. L., Miller K. D., Jemal A. (2017). Cancer statistics, 2017. *CA: A Cancer Journal for Clinicians*.

[2] Torre L. A., Siegel R. L., Jemal A. (2016). Lung cancer statistics. *Lung Cancer and Personalized Medicine*.

[3] Doi K. (2007). Computer-aided diagnosis in medical imaging: historical review, current status and future potential. *Computerized Medical Imaging and Graphics*.

[4] Song Q., Zhao L., Luo X., Dou X. (2017). Using deep learning for classification of lung nodules on computed tomography images. *Journal of Healthcare Engineering*.

[5] Lin T.-Y., Goyal P., Girshick R., He K., Dollár P. Focal loss for dense object detection.

[6] Lecun Y., Bottou L., Bengio Y., Haffner P. (1998). Gradient-based learning applied to document recognition. *Proceedings of the IEEE*.

[7] Krizhevsky A., Sutskever I., Hinton G. E. ImageNet classification with deep convolutional neural networks.

[8] Simonyan K., Zisserman A. Very deep convolutional networks for large-scale image recognition.

[9] He K., Zhang X., Ren S., Sun J. Deep residual learning for image recognition.

[10] Li W., Cao P., Zhao D., Wang J. (2016). Pulmonary nodule classification with deep convolutional neural networks on computed tomography images. *Computational and Mathematical Methods in Medicine*.

[11] Kuruvilla J., Gunavathi K. (2014). Lung cancer classification using neural networks for CT images. *Computer Methods and Programs in Biomedicine*.

[12] Choi W.-J., Choi T.-S. (2013). Automated pulmonary nodule detection system in computed tomography images: a hierarchical block classification approach. *Entropy*.

[13] Setio A. A. A., Ciompi F., Litjens G. (2016). Pulmonary nodule detection in CT images: false positive reduction using multi-view convolutional networks. *IEEE Transactions on Medical Imaging*.

[14] Torres E. L., Fiorina E., Pennazio F. (2015). Large scale validation of the M5L lung CAD on heterogeneous CT datasets. *Medical Physics*.

[15] Setio A. A. A., Traverso A., de Bel T. (2017). Validation, comparison, and combination of algorithms for automatic detection of pulmonary nodules in computed tomography images: the LUNA16 challenge. *Medical Image Analysis*.

[16] Armato S. G., McLennan G., Bidaut L. (2011). The lung image database consortium (LIDC) and image database resource initiative (IDRI): a completed reference database of lung nodules on ct scans. *Medical Physics*.

[17] Manos D., Seely J. M., Taylor J., Borgaonkar J., Roberts H. C., Mayo J. R. (2014). The lung reporting and data system (LU-RADS): a proposal for computed tomography screening. *Canadian Association of Radiologists Journal*.

[18] Naidich D. P., Bankier A. A., MacMahon H. (2013). Recommendations for the management of subsolid pulmonary nodules detected at CT: a statement from the Fleischner society. *Radiology*.

[19] Murphy K., van Ginneken B., Schilham A. M. R., de Hoop B. J., Gietema H. A., Prokop M. (2009). A large-scale evaluation of automatic pulmonary nodule detection in chest CT using local image features and k-nearest-neighbour classification. *Medical Image Analysis*.

[20] Jacobs C., van Rikxoort E. M., Twellmann T. (2014). Automatic detection of subsolid pulmonary nodules in thoracic computed tomography images. *Medical Image Analysis*.

[21] Setio A. A. A., Jacobs C., Gelderblom J., van Ginneken B. (2015). Automatic detection of large pulmonary solid nodules in thoracic CT images. *Medical Physics*.

[22] de Boer P.-T., Kroese D. P., Mannor S., Rubinstein R. Y. (2005). A tutorial on the cross-entropy method. *Annals of Operations Research*.

[23] Kinga D., Adam J. B. A method for stochastic optimization.

